# Parent Satisfaction With the Electronic Medical Record in an Academic Pediatric Rheumatology Practice

**DOI:** 10.2196/jmir.1525

**Published:** 2011-05-27

**Authors:** Paul Rosen, Steven J Spalding, Michael J Hannon, Robert M Boudreau, C Kent Kwoh

**Affiliations:** ^5^Department of MedicineUniversity of PittsburghPittsburgh VA Healthcare SystemPittsburgh, PAUnited States; ^4^Graduate School of Public HealthUniversity of PittsburghPittsburgh, PAUnited States; ^3^Department of MedicineUniversity of PittsburghPittsburgh, PAUnited States; ^2^The Cleveland ClinicCleveland, OHUnited States; ^1^Children's Hospital of Pittsburgh of UPMCUniversity of Pittsburgh School of MedicinePittsburgh, PAUnited States

**Keywords:** Electronic medical record, pediatric rheumatology, ambulatory care

## Abstract

**Background:**

Patient satisfaction has not been widely studied with respect to implementation of the electronic medical record (EMR). There are few reports of the impact of the EMR in pediatrics.

**Objective:**

The objective of this study was to assess the impact of implementation of an electronic medical record system on families in an academic pediatric rheumatology practice.

**Methods:**

Families were surveyed 1 month pre-EMR implementation and 3 months post-EMR implementation.

**Results:**

Overall, EMR was well received by families. Compared with the paper chart, parents agreed the EMR improved the quality of doctor care (55% or 59/107 vs 26% or 26/99, P < .001). More parents indicated they would prefer their pediatric physicians to use an EMR (68% or 73/107 vs 51% or 50/99, P = .01).

**Conclusions:**

Transitioning an academic pediatric rheumatology practice to an EMR can increase family satisfaction with the office visit.

## Introduction

Implementation of the electronic medical record (EMR) is currently taking place across industrialized countries. In 1991, the Institute of Medicine (IOM) issued a report concluding that computer-based patient records were an “essential technology” for health care and in 1997 called for the widespread adoption of a computer-based patient record over the next 10 years [1]. Little work has been done to study the direct influence of information technology on patient-physician relationships [2].

The 2001 IOM report, *Crossing the Quality Chasm: A Hew Health System for the 21st Century*, targeted six areas of health care that required significant improvement: safety, efficacy, timeliness, efficiency, equality, and patient-centeredness [3]. The report delineated how health information technology (HIT) was necessary to achieve all six aims. The United States government has recently reinforced the call issued for further adoption of HIT in the IOM report with the allocation of more than US $20 billion in the American Recovery and Reinvestment Act of 2009. However, some have argued that the use of HIT can hurt the delivery of patient-centered care [4-7].

Widespread EMR adoption has been slow. In 2008, DesRoches and colleagues surveyed over 2700 physicians and found that the implementation rate of a fully functional electronic medical record (EMR) system was 4%. Another 13% of physicians were using a more basic electronic medical system with limited functionality [8].

Few studies have examined the impact of EMR implementation in the pediatric setting, and, to our knowledge, none have looked at its impact on parent satisfaction and the doctor-patient relationship in ambulatory subspecialty pediatrics. At our academic children’s hospital, ambulatory offices started conversion to an EMR in 2005. While all 20 ambulatory practices converted to the EMR by 2009, we were the first practice to make the conversion. Our study was designed to determine if the transition to EMR was associated with changing parent satisfaction.

## Methods

This study was conducted at an academic pediatric rheumatology practice in Pittsburgh, Pennsylvania. The practice conducts over 95% of its patient visits in the ambulatory setting and has a small inpatient hospital service. The outpatient practice adopted a complete electronic medical record (Cerner PowerChart Office, Cerner Corporation, Kansas City, MO)

Prior to the transition to the EMR, the physicians worked with Cerner programming staff to design a universal rheumatology template that could be used for all outpatient rheumatology visits. The components of the computerized note template that were unique to pediatric rheumatology included a complete joint examination in the physical examination section. There was also a screening section that served as a reminder to the physicians to document routine pediatric rheumatology screening such as completion of eye examinations for uveitis, influenza vaccinations for immunosuppressed patients, and purified protein derivative (PPD) status. Physicians worked with the technical staff to develop the template and test the final note prior to implementation. The differences in the workflow between the pre-EMR paper charts and the post-EMR implementation are illustrated in Table 1.

**Table 1:** Comparison of office practices pre- and post-EMR implementation

Office Practice	Pre-EMR (Paper Charts)	EMR
Office visit letters	Physician dictates via telephone	Physician creates Cerner PowerNote via computer
Lag time for letter to be sent to referring physician	Letter faxed 3 to 5 business days after dictation	EMR note faxed immediately upon completion and signature
Prescription format	Hand written	EMR-generated via Cerner EZScript
Billing	Hand written on a form by physician and then entered electronically by secretary	Entered electronically by physician
Orders for laboratory tests, diagnostic tests, radiographs, consultations, etc	Hand written on forms or prescriptions	Entered electronically by physician with EMR-generated paper form for patient
Messaging between medical staff members	Paper slip attached to paper chart	Electronic messaging in patient’s chart via electronic in-box
Laboratory, pathology, and radiograph result endorsement by physician	Result on paper initialed	Electronic endorsement via electronic in-box
Results or other medical records from outside hospitals	Papers manually added to paper charts	Electronically scanned into EMR

Computers were placed in all six patient examination rooms. In all, three computers were placed in the common work area for the use of 3 attending physicians, 2 nurses, and trainees (fellows and residents). Computers and printers were installed with existing office furniture. The layout of the examination rooms was not changed to accommodate the new workflow.

Physicians received three training sessions (one hour each) on EMR usage and specifically on how to integrate computers into the visit. On-site technical support was available during the 6-week implementation phase. Support by telephone was provided after that. The patient schedule was reduced by 50% for the first 2 weeks of EMR implementation. The schedule was then reduced by 25% for the following 4 weeks of EMR implementation. During the 6 weeks that the schedule was reduced, there was no change in time allotment for each patient appointment.

### Patients

Participants were the parents of the patients of two of the authors. The patients were children with diagnoses typical of a pediatric rheumatology practice including juvenile idiopathic arthritis, fibromyalgia, systemic lupus erythematosus, dermatomyositis, scleroderma, vasculitis, and other chronic autoimmune conditions. This was a convenience sample, and all families coming to the office for their routine follow-up visits were asked to complete a parent survey after their visit was completed. All of the parents approached by the investigators agreed to complete the surveys.

For the 1-month period prior to transitioning to the EMR, we conducted surveys of the parents of all of our follow-up patients regarding the paper medical record. Starting 3 months after adoption of the EMR, for the next month families coming for their routine follow-up visits were surveyed. Names of the parents completing the surveys were not recorded so the number of patients who overlapped for the pre- and post-EMR implementation surveys could not be determined.

The parent surveys included 12 statements about the medical record (paper or electronic depending on the time point), quality of care received, family satisfaction with the office visit, and patient safety. The parents recorded their level of agreement with each statement on a 5-point Likert scale from *strongly agree* to *strongly disagree*. Of the 12 statements, 2 assessed computer usage (on a 5-point scale from *never* to 5 hours a day) and skill (on a 5-point scale from *poor* to *excellent*).

The study was submitted to the University of Pittsburgh institutional review board (IRB). The study was reviewed by the IRB, and it was determined that the project was primarily a quality assurance activity. The study was sent to two quality committees for review. The study was approved as a quality improvement study by the Children’s Hospital of Pittsburgh Total Quality Council and by the University of Pittsburgh Medical Center Quality Assurance Committee.

### Statistical Analysis

Because of the distribution of the responses, the survey responses were dichotomized into two groups: strongly agree/agree and strongly disagree/disagree/neutral prior to analysis. Fisher’s exact test was used to compare responses for pre- and post-EMR implementation. Spearman correlation coefficients (ρ’s) were calculated to evaluate the family’s view of quality of care and the doctor-patient relationship after EMR implementation. Correlations were based on post hoc observations and analyses; they were not hypothesis driven. Correlation coefficients of .3 to .5 were considered weak; .5 to .7, moderate; and greater than .7, strong. Statistical Analysis Software (SAS Institute Inc, Cary, NC) was used to perform statistical analysis.

## Results

Overall, families reported greater satisfaction with the EMR compared with the paper chart (Table 2).

**Table 2:** Parent survey of medical record pre- and 3 months post-EMR implementation

	Paper Chart, *Strongly Agree* and *Agree* (n = 99)	Electronic Medical Record, *Strongly * *agree* and *agree* (n = 107)	*P* value^a^
Statement	n (%)	n (%)	
1. The current medical record system increases the time the doctor spends with my child.	58 (59)	62 (58)	.99
2. I would like more of my child’s physicians to use an electronic medical record system.	50 (51)	73 (68)	.01
3. I would *not* miss the current charting system if it was no longer available.	53 (54)	10 (9)	< .001
4. I am dissatisfied with the current charting system used by my child’s doctor.	6 (6)	2 (2)	.16
5. I worry that my child’s private medical chart may be seen by others.	22 (22)	23 (21)	.99
6. The charting system allows me to better communicate with my child’s doctor.	45 (45)	56 (53)	.33
7. The current charting system helps me to understand my child’s medical tests.	33 (33)	51 (48)	.047
8. The current medical record system improves the quality of care provided by my child’s doctor.	26 (26)	59 (55)	< .001
9. I feel that the current medical record system distances me from my child’s doctor.	5 (5)	4 (4)	.74
10. I feel that the current medical record system adequately prevents medical errors.	14 (14)	14 (13)	.84
11. The information in my child’s chart is kept current.	69 (70)	89 (83)	.03
12. The staff have adequately addressed my concerns about the current medical record system.	39 (39)	73 (68)	< .001

^a^ Based on Fisher’s exact test

Parents rated the quality of care provided by the doctor using the EMR higher compared with the paper chart (*P* < .001). Parents felt that the information in the EMR was up-to-date compared with the paper chart (*P* = .03); this aspect of the EMR had the highest percentage of parents responding *agree/strongly agree* (83% or 89/107). In addition, parents felt that the EMR helped them to understand their child’s medical tests compared with the paper chart (*P* = .05).

Parents reported that they would like more of their child’s physicians to use an EMR (*P* = .01). The parents’ desire for other physicians to use EMR correlated with reporting more time spent with the doctor (ρ = 0.52, *P* < .001), better communication with the doctor (ρ = 0.50, *P* < .001), better understanding of medical tests (ρ = 0.42, *P* < .001), and higher quality of care (ρ = 0.41, *P* < .001). Family satisfaction with physician communication correlated with reporting better understanding of medical tests (ρ = 0.67, *P* < .001) and improved quality of care (ρ = 0.51, *P* < .001).

The majority of parents reported better than average computer skills and more than 5 hours of computer usage a month.

## Discussion

This study demonstrates the impact on families of implementation of an electronic medical record in an academic pediatric subspecialty practice. Parents indicated greater satisfaction with the EMR compared with paper charts. Parents reported a preference for their child’s other physicians to use an EMR.

When planning a conversion to an EMR, physicians may be concerned that the quality of the visit with families will be compromised. A 2006 report from Israel focusing on the EMR in the outpatient setting showed that physicians spent between 25% and 42% of the visit gazing at the computer screen [6]. In this observational study, monitor gazing decreased physician psychosocial questioning and emotional responsiveness to the patient. In addition, physician keyboarding decreased both physician and patient contribution to the medical dialogue [6]. In our study, physician computer usage in the examination room did not result in a negative patient perception.

Other reports have documented beneficial effects of an EMR. Using videotaped encounters, Arar et al showed that EMR use enhanced patient-physician communication and safety by facilitating medication reconciliation [9]. The group also found that use of an EMR, compared with a paper chart, was more likely to result in documentation of a diagnosis, of advice given, and of a referral ordered. In 2007, Simon et al surveyed a random sample of over 1000 physicians in Massachusetts and assessed physicians’ perceptions of the EMR in medical practice. They found that compared with physicians not using an EMR, physicians using an EMR reported greater patient-physician communication [10].

Our findings of improved family satisfaction with an EMR are similar to those reported in a longitudinal quantitative study on the impact of computers in ambulatory care on patient-physician interactions by Hsu et al in 2005 [11]. The group surveyed patients 2 months prior to EMR implementation, and 1 and 7 months after EMR implementation. By 7 months, the investigators found improvements in the following areas of patient satisfaction: overall visit, physician’s level of familiarity with the patient, communication about medical issues, and degree of comprehension with decisions made during the visit. In addition, the patients did not feel that there was less time for discussion about psychosocial issues with the computers in the exam room. There were no decreases in patient satisfaction areas such as physician’s personal manner, level of concern for the patient, and attention to listening [11].

In 2006, Kemper et al reported 21% of 1000 pediatricians responding to a survey had an electronic health record in their practice [12]. The group listed the perceived barriers to implementation among general pediatricians without an EMR. Barriers included: expense of implementation, lack of EMR that meets the needs of a pediatric practice, physician resistance, increase in physician workload, inadequate computer skills of providers, lack of improvement in patient care, interference with doctor-patient relationship, and concerns about patient confidentiality.

### Limitations

Our study faced several potential limitations. First, this study was conducted in an academic pediatric subspecialty office at a tertiary care children’s hospital thus limiting the application to the general pediatric physician workforce. Because the surveys were anonymous, we were not able to track participant overlap in pre- and post-EMR implementation surveys or compare response rate pre- and post-EMR for the exact same groups of patients. In addition, as early adopters of the technology, the physicians in our practice may not be representative of other pediatric subspecialists. In our study, the physicians caring for the patients also recruited the patients for the study. Thus, response bias among participants toward a parent’s perception of how the physicians felt about the EMR may have been introduced. In addition, our use of convenience sampling may have lead to biased results. Because our surveys included the families of just two physicians, our results may not generalize to larger groups of physicians. Our results need to be replicated across broader settings with random survey recruitment. Future recruitments should separate the roles of clinician and study recruiter.

As part of a large institution, the rheumatology physicians did not experience the financial impact of the reduction in patient scheduling. In addition, there was no up-front capital investment required from the physicians in order to purchase, implement, and maintain the technology. This would not hold true for the majority of practicing pediatricians.

### Conclusions

Health care leaders, government officials, and policy makers have been calling for a paperless medical record system for over 2 decades. Despite a call for universal adoption of an EMR for all patients, implementation has been limited. In pediatrics, the challenges of implementing an EMR in a small practice are real. In our academic, hospital-supported, subspecialty practice we were able to transition to an EMR with a subsequent increase in patient satisfaction. More study in the area of EMR implementation in pediatrics is warranted**.**

## Abbreviations

EMR: electronic medical record
IOM: Institute of Medicine
IRB: institutional review board
HIT: health information technology
SAS: Statistical Analysis Software
PPD: purified protein derivative
